# Effect of Congruence Between Sound and Video on Heart Rate and Self-Reported Measures of Emotion

**DOI:** 10.5964/ejop.v14i3.1593

**Published:** 2018-08-31

**Authors:** Sebastián Calderón, Raúl Rincón, Andrés Araujo, Carlos Gantiva

**Affiliations:** a Department of Engineering, Universidad de San Buenaventura, Bogotá, Colombia; b Department of Psychology, Universidad de San Buenaventura, Bogotá, Colombia; Department of Psychology, Webster University Geneva, Geneva, Switzerland; University of South Wales, Newport, United Kingdom

**Keywords:** bimodal stimuli, congruence, heart rate, valence, arousal

## Abstract

Most studies of emotional responses have used unimodal stimuli (e.g., pictures or sounds) or congruent bimodal stimuli (e.g., video clips with sound), but little is known about the emotional response to incongruent bimodal stimuli. The aim of the present study was to evaluate the effect of congruence between auditory and visual bimodal stimuli on heart rate and self-reported measures of emotional dimension, valence and arousal. Subjects listened to pleasant, neutral, and unpleasant sounds, accompanied by videos with and without content congruence, and heart rate was recorded. Dimensions of valence and arousal of each bimodal stimulus were then self-reported. The results showed that heart rate depends of the valence of the sounds but not of the congruence of the bimodal stimuli. The valence and arousal scores changed depending on the congruence of the bimodal stimuli. These results suggest that the congruence of bimodal stimuli affects the subjective perception of emotion.

Emotion is a prerequisite for action that results from the activation of one of two primary motivational systems (i.e., appetitive and defensive) by the perception of a stimulus (Lang, 1995). When the appetitive motivational system is activated, approach behaviors are generated, with the goal of consummation, procreation, or nurturance. Conversely, when the defensive motivational system is activated, defense behaviors to protect the organism from threat are generated. The activation of these two systems modulates reflex responses (e.g., heart rate) that are compatible or incompatible with the required behavior (Alpers, Adolph, & Pauli, 2011). Emotional reactions to external cues are modulated by valence and arousal dimensions. Valence dimension (i.e., pleasure or displeasure) is related to the active motivational system, and arousal dimension is related to the intensity of emotional response (Bradley, Codispoti, Cuthbert, & Lang, 2001). The heart rate response is one of the most used physiological measures in studies of emotion (Barry, 1984; Berntson, Quigley, & Lozano, 2007; Lang, 1995; Obrist, 1981; Turpin, 1986). Heart rate is sensitive to the interaction between valence and arousal (Bradley et al., 2001) and is a reliable indicator of attentional engagement that is generated by the stimuli (Bradley, 2009; Campbell, Wood, & McBride, 1997).

Previous studies have reported the effects of affective stimulus presentation on heart rate. Bradley et al. (2001), found a pattern of initial deceleration of heart rate during the presentation of affective pictures. Higher deceleration occurred in response to unpleasant and pleasant pictures with high arousal compared with neutral pictures. Similar results were found with the presentation of affective sounds (i.e., initial deceleration, followed by acceleration and deceleration). Unpleasant sounds that evoked high arousal generated greater initial heart rate deceleration than pleasant sounds that evoked high arousal and neutral sounds (Bradley & Lang, 2000). These results suggest that initial heart rate deceleration occurs as a result of the response of attention that is generated by the interaction between valence and arousal and parasympathetic dominance of the autonomic nervous system (Bradley & Lang, 2007a).

Studies with motion pictures compared with still pictures have reported greater heart rate deceleration while viewing motion pictures, and movement affected self-reported valence and arousal (Detenber, Simons, & Bennett, 1998; Simons, Detenber, Roedema, & Reiss, 1999). Studies with videos found greater heart rate deceleration in response to unpleasant film clips compared with pleasant and neutral film clips (Bos, Jentgens, Beckers, & Kindt, 2013; Codispoti, Surcinelli, & Baldaro, 2008; Gomez, Zimmermann, Guttormsen-Schär, & Danuser, 2005). However, when the pictures contained a phobic stimulus (e.g., snakes or spiders; Hamm, Cuthbert, Globisch, & Vaitl, 1997) or when the videos contained an explicit threat or a phobic stimulus (Courtney, Dawson, Schell, Iyer, & Parsons, 2010; Palomba, Sarlo, Angrilli, Mini, & Stegagno, 2000), initial heart rate acceleration was observed, consistent with a sympathetic response of the autonomic nervous system.

In everyday life, emotional experience is often influenced by both visual and auditory information. The bimodal integration of these stimuli is important for understanding the emotional experience. Studies of emotional responses to bimodal stimuli have been performed using self-report measures and physiological responses. For example, Cox (2008) used self-report measures to evaluate the interaction between unpleasant sounds and pictures that were either associated or not associated with the sounds. This author found that pictures that were associated with sounds (with the exception of disgusting sounds) increased the level of sound aversion in some cases. Scherer and Larsen (2011) evaluated the effect of cross-modal priming, a phenomenon that occurs when a prime that is presented in one sensory modality (e.g., auditory) influences responses to a target in a different sensory modality (e.g., visual). They used affective sounds (i.e., pleasant and unpleasant) as a prime for affective visual stimuli (i.e., pleasant and unpleasant words) and found significant cross-modal priming effects but only for unpleasant primes (i.e., the participants evaluated the positive words as negative after listening to an unpleasant sound).

Studies that have used electrophysiological measures showed that event-related potential (P100 and P200) amplitudes increased in response to all types of pictures (i.e., pleasant, neutral, and unpleasant) that were accompanied by emotional sounds compared with pictures that were accompanied by neutral sounds (Gerdes et al., 2013). Brouwer, van Wouwe, Mühl, van Erp, and Toet (2013) presented unimodal and bimodal stimuli that were composed of affective pictures and sounds. Their results showed no differences in heart rate, heart rate variability, galvanic skin response, or self-report measures in response to pleasant and unpleasant stimuli, independent of the modality (i.e., unimodal or bimodal). The authors recognized that these results may be attributable to the fact that the bimodal stimuli were not optimally congruent.

Neuroimaging studies have shown that the processing of incongruent bimodal stimuli requires greater cognitive control, reflected by activation of the inferior frontal cortex. The processing of congruent bimodal stimuli was shown to activate temporal regions (Belardinelli et al., 2004; van Atteveldt, Formisano, Goebel, & Blomert, 2007). From a functional perspective, these data imply a greater investment of cognitive resources to evaluate incongruent bimodal stimuli compared with congruent bimodal stimuli (Doehrmann & Naumer, 2008).

Research that evaluates congruence between bimodal stimuli has been limited because only sounds and pictures are typically used (Gerdes, Wieser, & Alpers, 2014), thus generating less of an emotional response than videos (Courtney et al., 2010; Detenber et al., 1998; Simons et al., 1999). Such bimodal stimuli (pictures and sounds) are also less familiar compared with everyday experiences, which often contain movement stimuli. Additionally, the most commonly used affective stimuli are pictures that are contained in the International Affective Picture System (IAPS; Lang, Bradley, & Cuthbert, 2008) and sounds that are contained in the International Affective Digitized Sound System (IADS; Bradley & Lang, 2007b). Finding absolute congruence between these two types of stimuli is very difficult because each stimulus group was built independently (i.e., the IAPS has over 1000 pictures, and the IADS has 167 sounds). Furthermore, the sounds are dynamic, and the pictures are static, which limits evaluations of the effects of congruence between bimodal stimuli.

The objective of the present study was to evaluate the effect of congruence between bimodal stimuli that were composed of emotional sounds in the IADS (Bradley & Lang, 2007b) and videos on heart rate and self-report measures of valence and arousal. Our hypothesis was that congruent bimodal stimuli that are more realistic and captivating than incongruent bimodal stimuli (because they make more sense to the subject) would have a stronger emotional impact, reflected by subjective measures. In contrast, incongruent bimodal stimuli require a higher workload to be processed and thus would generate heart rate acceleration. The results of this study will provide evidence of the mechanisms by which information from the environment is integrated.

## Methods

### Participants

Thirty right-handed undergraduate students (24 males and six females), ranging in age from 20 to 27 years (*M* = 22.67 years, *SD* = 2.26 years), participated in the study. The participants were recruited from a university in Bogota, Colombia. The exclusion criteria were current medical or psychological treatment and visual/auditory problems without correction. All of the participants provided written informed consent. The study was approved by the University of San Buenaventura Review Board.

### Stimuli and Procedure

Thirty sounds (10 pleasant with high arousal, 10 neutral with low arousal, and 10 unpleasant with high arousal) were selected from the IADS^i^ according to normative ratings that were proposed by Bradley and Lang (2007b). Significant differences were found between sound categories in the valence dimension (all *p* < .001). For the arousal dimension, significant differences were found between affective sounds (pleasant and unpleasant) and neutral sounds (all *p* = .001). Pleasant and unpleasant sounds were not significantly different in the arousal dimension (*p* > .05). Sixty videos were selected from different web pages (e.g., YouTube or Google videos). Half of the videos had high congruence with the selected IADS sounds, and the other half had no congruence.

To obtain bimodal stimuli, videos with and without content congruence were added to each sound. Bimodal stimuli with congruence had credibility between the sources of the sound and video (e.g., for the sound of a roller coaster going downhill, the congruent video showed a rollercoaster going *downhill*). In contrast, bimodal stimuli without congruence had no credibility between the sources of the sound and video (e.g., for the sound of a roller coaster going downhill, the incongruent video showed a roller coaster going *uphill*). Before the experiment, each bimodal stimulus was evaluated by 52 subjects on a scale of 1 to 9 (1 = complete non-congruence, and 9 = complete congruence). Congruent bimodal stimuli had a mean score of 8.03 (*SD* = .40). Incongruent bimodal stimuli had a mean score of 1.55 (*SD* = .75). None of the subjects who participated in the initial evaluation of the bimodal stimuli participated in the experiment. Sounds and videos were edited using Adobe Premiere Pro CC software. Each bimodal stimulus pair was presented for 6 s at a resolution of 1280 × 720 pixels and frame rate of 24 frames per second.

The 60 bimodal stimuli were presented on a 19-inch flat-screen monitor that was located approximately 60 cm from the subject and through headphones. The sound level was 65 dB. Two different pseudo-randomized bimodal stimulus presentation orders were prepared (each order was applied to 15 subjects). Each order had the constraint of not presenting the same sound category consecutively and not presenting congruent or incongruent bimodal stimuli consecutively. Each bimodal stimulus pair was presented once. After each bimodal stimulus pair presentation, the emotional experience of each participant (i.e., valence and arousal ratings) was evaluated using digital versions of the Self-Assessment Manikin (SAM; Bradley & Lang, 1994). The intertrial interval (ITI) varied randomly from 10 to 14 s.

### Self-Report Measures

Valence and arousal ratings were obtained using digitized versions of the SAM (Bradley & Lang, 1994), which depicts a graphic figure that varies along two dimensions of pleasure and arousal on a 9-point scale. The SAM ranges from smiling and happy (9) to frowning and unhappy (1) when representing the valence dimension. For the arousal dimension, the SAM ranges from excited and wide eyed (9) to relaxed and sleepy (1).

### Data Acquisition and Reduction

The bimodal stimuli were presented using E-Prime 2.0 software (Psychology Software Tools, PA, USA). Electrocardiogram (EKG) recordings were continuously collected at a sampling rate of 1000 Hz with a 10-500 Hz frequency band filter using PowerLab 26T equipment (ADInstruments, Australia) using three large Sensormedic electrodes filled with electrolyte paste at lead III. All of the EKG data was manually inspected prior to analysis. Heart rate was derived from the EKG. R-wave–to–R-wave (R-R) intervals were extracted from the EKG using LabChart 7.3 software (ADInstruments, Australia), which were then imported into Matlab 8.0 (MathWorks, MA, USA) for analysis. Heart rate responses were determined by averaging across each half-second during the 6-s bimodal stimulus presentation and subtracting that activity from the activity that was obtained 1 s before bimodal stimulus onset.

### Statistical Analysis

Heart rate responses were analyzed using a 3 × 2 × 12 repeated-measures analysis of variance (RM-ANOVA), with Sound (pleasant, neutral, and unpleasant), Video Congruence (with and without), and Time (the 12 half-second time bins throughout the duration of bimodal stimulus presentation) as the repeated measures. SAM ratings were analyzed using a 3 × 2 ANOVA, with Sound and Video Congruence as the repeated measures. Greenhouse-Geisser correction was applied to correct any violation of sphericity. *Post hoc* analyses of mean values were performed using the paired multiple-comparison Least Significant Difference (LSD) test. The level of significance for all of the analyses was *p* < .05. The partial eta squared (η^2^_p_) is also presented. All of the analyses were performed using SPSS 20.0 software.

## Results

### Heart Rate Response

The ANOVA revealed a significant main effect of Time (*F*(11, 319) = 3.689, *p* = .041, η^2^_p_ = .11). We identified two deceleration phases: one phase between 0.5 and 1.5 s (with significant differences between 0.5 s and 1 and 1.5 s; both *p* < .02) and one phase between 3 and 5.5 s (with significant differences between 3 s and 5 and 5.5 s; both *p* < .02). Significant differences at all time points between 0.5 s and 3.5-6 s were found (all *p* < .05). A trend analysis was performed to identify the pattern of heart rate responses, showing a significant linear trend (*F*(1, 29) = 4.752, *p* = .038, η^2^_p_ = .14).

**Figure 1:**
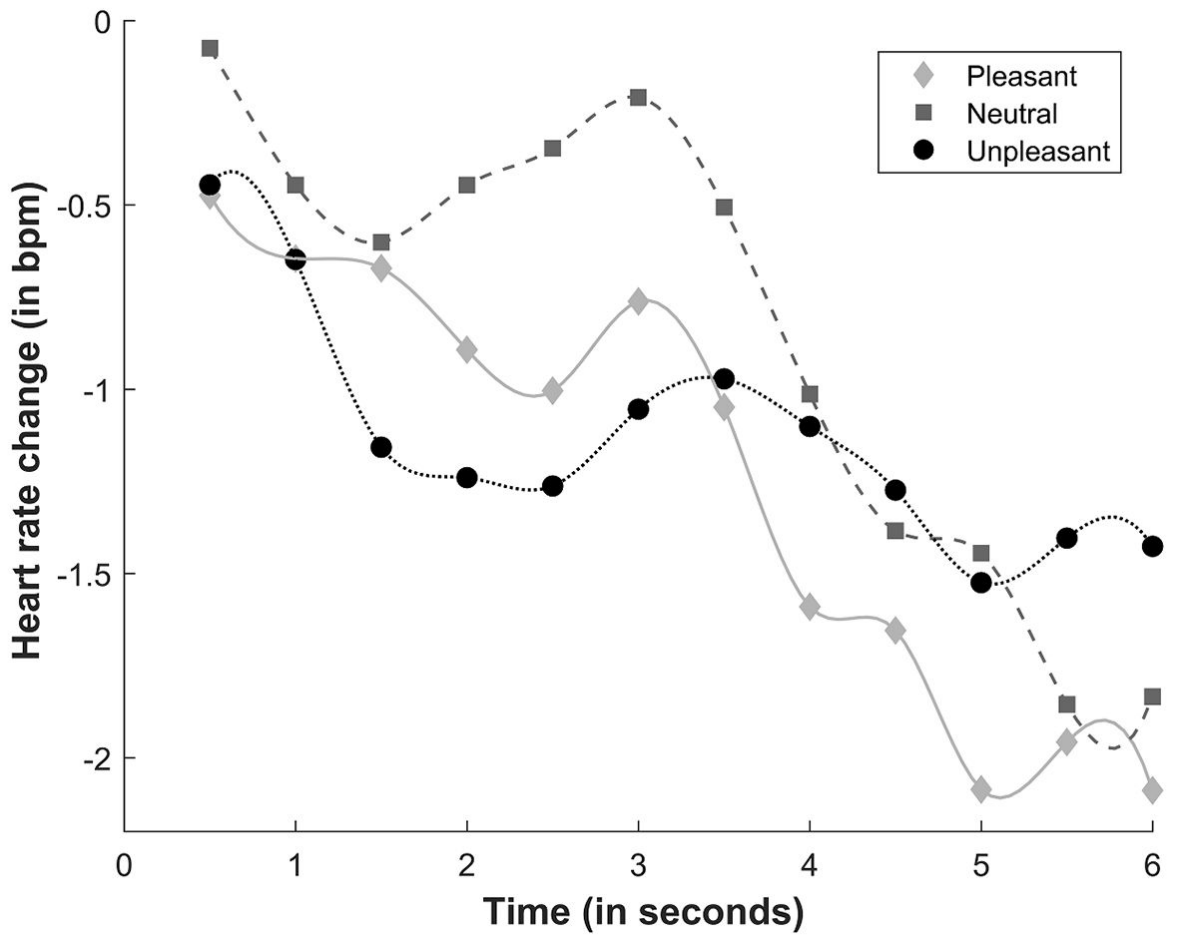
Heart rate response to sounds. bpm, beats per minute.

A significant Sound × Time interaction was found (*F*(22, 638) = 2.574, *p* = .049, η^2^_p_ = .08), suggesting that the valence of the bimodal stimuli generated differential heart rate response patterns at different time points. The deceleration of heart rate was greater during the presentation of unpleasant bimodal stimuli compared with neutral bimodal stimuli between 1.5 and 2.5 s (all *p* < .02). Similarly, the deceleration of heart rate during the presentation of pleasant sounds was greater compared with neutral sounds between 2 and 3 s (all *p* < .02). At 4.5 s, a significant acceleration of heart rate was found during the presentation of unpleasant sounds compared with pleasant sounds (*p* = .04; Figure 1). A significant linear trend was found for pleasant (*F*(1, 29) = 6.011, *p* = .02, η^2^_p_ = .17) and neutral (*F*(1, 29) = 9.761, *p* = .004, η^2^_p_ = .25) sounds. No significant main effects or interactions were found for Video Congruence (all *p* > .05).

### Subjective Ratings

#### Valence

**Figure 2:**
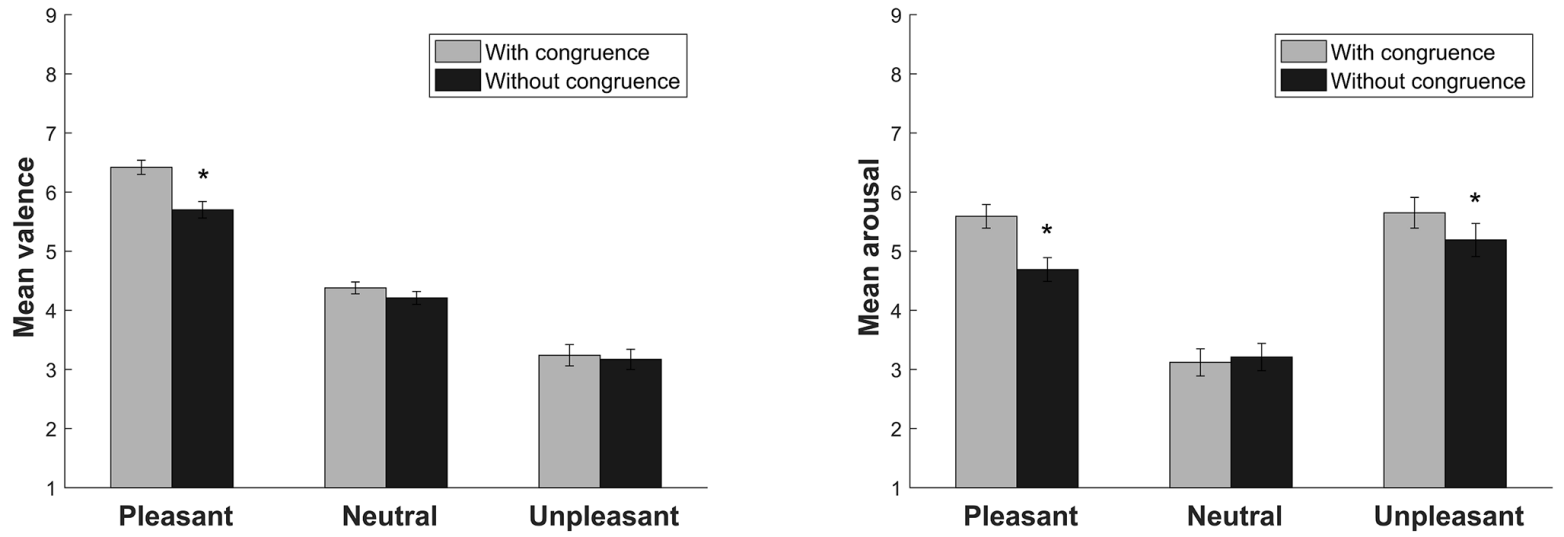
Subjective ratings of valence and arousal for the sounds with and without video congruence. Bars indicate the standard error of the mean. *p < .05.

The ANOVA of the valence dimension revealed a significant main effect of Sound (*F*(2, 58) = 132.41, *p* < .001, η^2^_p_ = .82). As expected, significant differences were found between pleasant, neutral, and unpleasant sounds (all *p* < .001). A significant main effect of Video Congruence was found (*F*(1, 29) = 15.13, *p* = .001, η^2^_p_ = .34). Sounds without video congruence had lower valence scores (*p* = .001). A significant Sound × Video Congruence interaction was found (*F*(2, 58) = 11.17, *p* < .001, η^2^_p_ = .27). Pleasant sounds were evaluated as less appetitive when they had no video congruence (*p* < .001; Figure 2).

#### Arousal

The ANOVA of the arousal dimension revealed a significant main effect of Sound (*F*(2, 58) = 75.04, *p* < .001, η^2^_p_ = .72). As expected, pleasant and unpleasant sounds were perceived as more arousing than neutral sounds (both *p* < .001). A significant main effect of Video Congruence was found (*F*(1, 29) = 22.52, *p* < .001, η^2^_p_ = .43). Sounds without video congruence were perceived as less arousing (*p* < .001). A significant Sound × Video Congruence interaction was found (*F*(2, 58) = 17.22, *p* < .001, η^2^_p_ = .37). Pleasant and unpleasant sounds without video congruence were perceived as less arousing than pleasant and unpleasant sounds with video congruence (both *p* < .002; Figure 2).

## Discussion

The objective of the present study was to evaluate the effect of congruence between bimodal stimuli (emotional sounds that are contained in the IADS and videos) on heart rate and self-reported measures of valence and arousal. The results suggested that the congruence between the sound and the video did not affect the heart rate response but affected the subjective evaluation of valence and arousal. Pleasant sounds without video congruence were evaluated as less appetitive, and pleasant and unpleasant sounds without video congruence were perceived as less arousing. These results suggest that the absence of congruence between bimodal stimuli changes the subjective emotional experience (i.e., it diminishes the level of arousal of the affective stimuli). In contrast, congruent stimuli are more real and captivating, leading to stronger emotional effects (i.e., greater arousal).

The absence of congruence also affected the valence of pleasant sounds but not the valence of unpleasant sounds. These data suggest a functional bias toward aversive stimulation because greater responsiveness to aversive stimuli than to appetitive stimuli is more adaptive for survival (Bradley & Lang, 2007a; Lang, Davis, & Öhman, 2000), independent of their congruence. Therefore, the valence of unpleasant sounds is more important than the congruence between auditory and visual stimulation. This bias toward aversive stimuli has been found in several studies that used affective pictures (Bradley et al., 2001; Gantiva & Camacho, 2016; Gantiva, Guerra, & Vila, 2011; Vila et al., 2001) and affective sounds (Bradley & Lang, 2000; Fernández-Abascal et al., 2008).

Congruence did not significantly affect heart rate, but a greater initial deceleration was observed in response to pleasant and unpleasant sounds compared with neutral sounds, indicating a greater attention response that was caused by the interaction between valence and arousal and dominance of the parasympathetic autonomic nervous system (Bradley & Lang, 2007a), at least during the first phase of bimodal stimulus presentation. Similar results have been reported by studies that used pictures (Bradley et al., 2001), videos (Bos et al., 2013; Codispoti et al., 2008; Gomez et al., 2005), and affective sounds (Bradley & Lang, 2000).

However, the unpleasant sounds subsequently generated an acceleration of heart rate. This acceleration was not observed when pleasant and neutral sounds were presented, indicating activation of the sympathetic autonomic nervous system that was associated with a motivational and behavioral response (Courtney et al., 2010; Palomba et al., 2000). Nevertheless, pleasant and neutral sounds maintained a relatively constant deceleration of heart rate.

The present results suggest that the congruence of bimodal stimuli increases the subjective emotional response, especially the perception of arousal. However, contrary to our hypothesis, the absence of congruence between bimodal stimuli did not increase heart rate. Similar results were previously found by Brouwer et al. (2013), who used bimodal stimuli that consisted of pictures and sounds. This suggests that the cardiac response is more influenced by the valence of the stimuli than by the degree of congruence, suggesting greater behavioral relevance of valence compared with congruence.

Several previous studies have shown the influence of valence on heart rate, in which aversive stimuli generated greater heart rate deceleration compared with appetitive and neutral stimuli (Bradley et al., 2001; Bradley & Lang, 2000). In fact, sustained heart rate deceleration is the most common initial response to a threatening stimulus (Campbell et al., 1997). According to the defense cascade model (Lang, Bradley, & Cuthbert, 1997), defensive responses begin with a stage of oriented attention (e.g., distant threat) and progress to a stage of defensive action (e.g., imminent attack). Heart rate deceleration is a characteristic physiological response of the first stage.

In conclusion, the present results suggest that incongruent bimodal stimuli affect subjective emotional experiences (i.e., cognitive processing), decreasing arousal perception and appetitive valence in the case of pleasant stimuli. This could happen because the cognitive processing of incongruent bimodal stimuli is more demanding than the processing of congruent stimuli (Gerdes et al., 2014; Gerdes et al., 2013). This implies a greater investment in cognitive processing while interpreting incongruent bimodal stimuli, thus leaving fewer resources for interpreting emotional subjective experiences. Conversely, congruence between stimuli does not require additional investments in cognitive resources, thus generating a greater subjective emotional impact.

In the present study, the cardiac response was unaffected by the congruence of stimuli, but it was affected by valence. This suggests that with regard to the physiological component of the emotional response (e.g., heart rate), the valence of the stimulus prevails over congruence. Nonetheless, congruence later modulates the perception of the emotional response. This corroborates the defense cascade model, in which physiological emotional reactions are a function of the valence of the stimulus (Lang et al., 1997). For example, when a stimulus that indicates a threat is perceived, heart rate initially decelerates, the skin conductance response increases, and the startle reflex is enhanced. These responses are generated to favor a fight or flight response and subsequent survival of the individual (Bradley & Lang, 2007a).

The relevance of our findings should be evaluated while considering several possible limitations. First, the sample did not include a balanced number of men and women. Similar studies should be conducted with broader samples and a balanced distribution of men and women, which would allow comparisons between sexes. Second, our study only revealed an effect of congruence of bimodal stimuli on heart rate responses and self-reported valence and arousal. Future studies should use other physiological measures to increase our understanding of the impact of the congruence of bimodal stimuli on the emotional response.
